# Facilitators and barriers of implementing end-of-life care volunteering in a hospital in five European countries: the iLIVE study

**DOI:** 10.1186/s12904-024-01423-5

**Published:** 2024-04-02

**Authors:** Berivan Yildiz, Agnes van der Heide, Misa Bakan, Grethe Skorpen Iversen, Dagny Faksvåg Haugen, Tamsin McGlinchey, Ruthmarijke Smeding, John Ellershaw, Claudia Fischer, Judit Simon, Eva Vibora-Martin, Inmaculada Ruiz-Torreras, Anne Goossensen, the iLIVE consortium, Simon Allan, Pilar Barnestein-Fonseca, Mark Boughey, Andri Christen, Nora Lüthi, Martina Egloff, Steffen Eychmüller, Sofia C. Zambrano, Gustavo G. De Simone, Eline E. C. M. Elsten, Eric C. T. Geijteman, Iris Pot, Carin C. D. van der Rijt, Carl Johan Fürst, Birgit H. Rasmussen, Maria E. C. Schelin, Christel Hedman, Gabriel Goldraij, Svandis Iris Halfdanardottir, Valgerdur Sigurdardottir, Tanja Hoppe, Melanie Joshi, Julia Strupp, Raymond Voltz, Maria Luisa Martín-Roselló, Silvi Montilla, Verónica I. Veloso, Vilma Tripodoro, Katrin Ruth Sigurdardottir, Hugo M. van der Kuy, Lia van Zuylen, Michael Berger, Rosemary Hughes, Hana Kodba-Ceh, Ida J. Korfage, Urska Lunder, Stephen Mason, Beth Morris, Kjersti Solvåg

**Affiliations:** 1 Department of Public Health, Erasmus MC, University Medical Center Rotterdam, Rotterdam, The Netherlands; 2 Research Department, University Clinic of Respiratory and Allergic Diseases Golnik, Golnik, Slovenia; 3 Regional Centre of Excellence for Palliative Care, Western Norway, Haukeland University Hospital, Bergen, Norway; 4 Department of Clinical Medicine K1, University of Bergen, Bergen, Norway; 5 Palliative Care Unit, Institute of Life Course and Medical Sciences, University of Liverpool, Liverpool, UK; 6 Department of Health Economics, Center for Public Health, Medical University of Vienna, Vienna, Austria; 7 CUDECA Institute for Training and Research in Palliative Care, CUDECA Hospice Foundation, Malaga, Spain; 8 Informal Care and Care Ethics, University of Humanistic Studies, Utrecht, The Netherlands

**Keywords:** End of life care, Volunteering, Implementation, Hospital volunteer, Inpatient

## Abstract

**Background:**

End-of-life (EoL) care volunteers in hospitals are a novel approach to support patients and their close ones. The iLIVE Volunteer Study supported hospital volunteer coordinators from five European countries to design and implement an EoL care volunteer service on general wards in their hospitals. This study aimed to identify and explore barriers and facilitators to the implementation of EoL care volunteer services in the five hospitals.

**Methods:**

Volunteer coordinators (VCs) from the Netherlands (NL), Norway (NO), Slovenia (SI), Spain (ES) and United Kingdom (UK) participated in a focus group interview and subsequent in-depth one-to-one interviews. A theory-inspired framework based on the five domains of the Consolidated Framework for Implementation Research (CFIR) was used for data collection and analysis. Results from the focus group were depicted in radar charts per hospital.

**Results:**

Barriers across all hospitals were the COVID-19 pandemic delaying the implementation process, and the lack of recognition of the added value of EoL care volunteers by hospital staff. Site-specific barriers were struggles with promoting the service in a highly structured setting with many stakeholders (NL), negative views among nurses on hospital volunteering (NL, NO), a lack of support from healthcare professionals and the management (SI, ES), and uncertainty about their role in implementation among VCs (ES). Site-specific facilitators were training of volunteers (NO, SI, NL), involving volunteers in promoting the service (NO), and education and awareness for healthcare professionals about the role and boundaries of volunteers (UK).

**Conclusion:**

Establishing a comprehensive EoL care volunteer service for patients in non-specialist palliative care wards involves multiple considerations including training, creating awareness and ensuring management support. Implementation requires involvement of stakeholders in a way that enables medical EoL care and volunteering to co-exist. Further research is needed to explore how trust and equal partnerships between volunteers and professional staff can be built and sustained.

**Trial registration:**

NCT04678310. Registered 21/12/2020.

**Supplementary Information:**

The online version contains supplementary material available at 10.1186/s12904-024-01423-5.

## Introduction

Over the past years, end-of-life (EoL) care volunteering has become an important contribution to high quality care for patients in their last phase of life [1]. EoL care volunteers have been shown to offer practical, emotional, social, and existential support in a way that improves the well-being of patients and their families [2, 3]. In almost every country in Europe, EoL care volunteers are actively engaged in hospices which would struggle to exist without their contributions in providing high quality care for dying patients [4]. Countries vary in the numbers and roles of volunteers, the tasks they perform and the developments in their organisations [5]. However, all do embrace “being there” as the core concept of this unique source of providing community care [6, 7]. In some countries, volunteers have had a long involvement in EoL care volunteering, while in other countries these services have only recently started.

A relatively uncommon setting of EoL care volunteering—even in countries with a long history of volunteering – is the hospital setting, and specifically wards not specialised in palliative care. It has been suggested that EoL care volunteers enable patients to maintain their “social capital” during their stay in the hospital and allow the process of dying not to be narrowed within a medical or solely professional context [8]. Studies have suggested that EoL care volunteer services have the potential to improve the experience of dying patients in the hospital and prevent loneliness, particularly for those without social networks [6, 9, 10]. Moreover, a recent systematic review showed that hospital palliative care volunteers were appreciated for providing psychosocial support, considered as complementary to, rather than replacing the work of health care professionals [11].

Hospital settings have different characteristics compared to other settings where volunteers may support patients in their last phase of life such as home, community or stand-alone hospices or palliative care volunteering services. This may imply a different nature of volunteering or factors leading to successful integration in hospital care [11]. Implementing a comprehensive EoL care volunteer service for patients in hospital wards, where the context and care culture are not per se focused on palliative care, entails the integration of various aspects including training, staffing and directing. So far, studies have predominantly focused on examining the experiences of volunteers, providing insight on the volunteer training requirements, and the difficulties and benefits of fulfilling the role of a volunteer in the hospital setting [12–14]. No studies are available about the experience of implementing an EoL care volunteer service in the hospital setting, from the perspective of those coordinating the implementation.

As part of the European Union Horizon 2020 funded iLIVE project [15], the iLIVE Volunteer study developed a European Core Curriculum (ECC) for EoL care volunteers in the hospital setting [16]. The curriculum includes specific attention to ensuring end-of-life-care volunteers are embedded within the organisation, including understanding the specific needs of wards within the hospital where the volunteers will be supporting dying patients [16]. The curriculum was used to train volunteers and establish an EoL care volunteer service for hospitalized patients in five hospitals (one hospital in each of the following countries: The Netherlands, Norway, Slovenia, Spain and United Kingdom). To better understand how an EoL care volunteer service can be implemented in the hospital setting, this study aims to identify barriers and facilitators as experienced during the implementation of EoL care volunteering in hospitals in the five countries.

## Methods

### Study design and setting

The five hospitals were part of the iLIVE Volunteer Study (Trial registration number NCT04678310) in which research staff had online international meetings on a regular basis. A timetable of the project with timeslots of data collection and data analysis is included in the supplementary file. In each of the five participating hospitals, a site-specific EoL care volunteer service was developed and implemented. The number of wards in which the volunteer services were offered ranged from 1 to 8. The volunteers were active during different time slots on working days and during weekends. (Table 1).

**Table 1:** Characteristics of the EoL care volunteer services in the five participating hospitals

	**Site A** The Netherlands	**Site B** Norway	**Site C** Slovenia	**Site D** Spain	**Site E** United Kingdom
**Characteristics of service**					
Active service time *(in months)* Service start date	24 April 2021	16 February 2022	16 August 2021	17 March 2022	16 November 2021
Total number of wards involved	8	7	2	1	1
Number of volunteers active in the service at start	12	13	5	7	17
Availability *(in days)*	7	5 (also on request during weekends and nights)	7	4	5
Timeslots available	10:00–22:00	08:30 – 19:00 (also nights on request)	15:00 – 18:00	12.00–17.00	9:00–17:00
E-volunteering offered	No	No	No	Yes	No
Frequency of provided supervision	Monthly	Bi-monthly	Monthly	Bi-monthly	Monthly
Number of volunteer coordinators	2	2	1	2	1
**Characteristics of hospital**					
Geographical characteristics	Large sized city with surroundings	City with surroundings	Rural region	Large sized city with surroundings	Large sized city with surroundings
General volunteer service active in hospital	Yes	Yes	No	No	Yes
**Number of patients supported**	47	13	10	103	203

### Participants and procedures

Volunteer coordinators (VC) were assigned to lead the development and implementation of the EoL care volunteer service in each hospital (Table 2). In the Netherlands, Norway and Spain, two VCs were appointed to coordinate the service in their hospitals; in the UK and Slovenia one VC was appointed. All VCs followed a three-day “Train-the-Trainer” course in the UK prior to developing and implementing their volunteer services. The aim of this course was to introduce the ECC and provide information and skills for the development and implementation of the EoL care services, including a focus on the development of a volunteer training.

**Table 2:** Characteristics of the VCs

	Age	Role within organization	Educational background	Participation to focus group	Participation to in-depth interview
VC A1	59	Volunteer coordinator	Kinesiology	Yes	Yes
VC A2	50	Volunteer coordinator	Management	Yes	No
VC B1	55	Volunteer coordinator, nurse	Palliative care nurse	Yes	Yes
VC B2	63	Coordinator communication department	TV production	No	Yes
VC C1	32	Voluntary services coordinator, researcher	Psychology	Yes	Yes
VC D1	42	Volunteer coordinator	Psychology	No	Yes
VC D2	29	Researcher	Psychology	Yes	No
VC E1	33	Voluntary services manager	Leadership and operational management	Yes	Yes

VCs were invited to participate to a focus group interview and an in-depth one-to-one interview about their experiences with implementation of the volunteer services. All VCs were female. VCs were contacted by e-mail and informed about the aims and procedure of both the focus group interview and one-to-one in-depth interview. Since the focus group interview took place as part of a project meeting, the researchers had also informed the VCs about practical and content-related details regarding the focus group interview. Most VCs had personally met the researchers before as part of the international project. All VCs explicitly provided verbal and written informed consent prior to participating in both the focus group interview and one-to-one in-depth interviews. The Consolidated criteria for reporting qualitative research (COREQ) checklist has been used to report necessary elements of the methods, analysis and results sections.

### Data collection

**Fig. 1:**
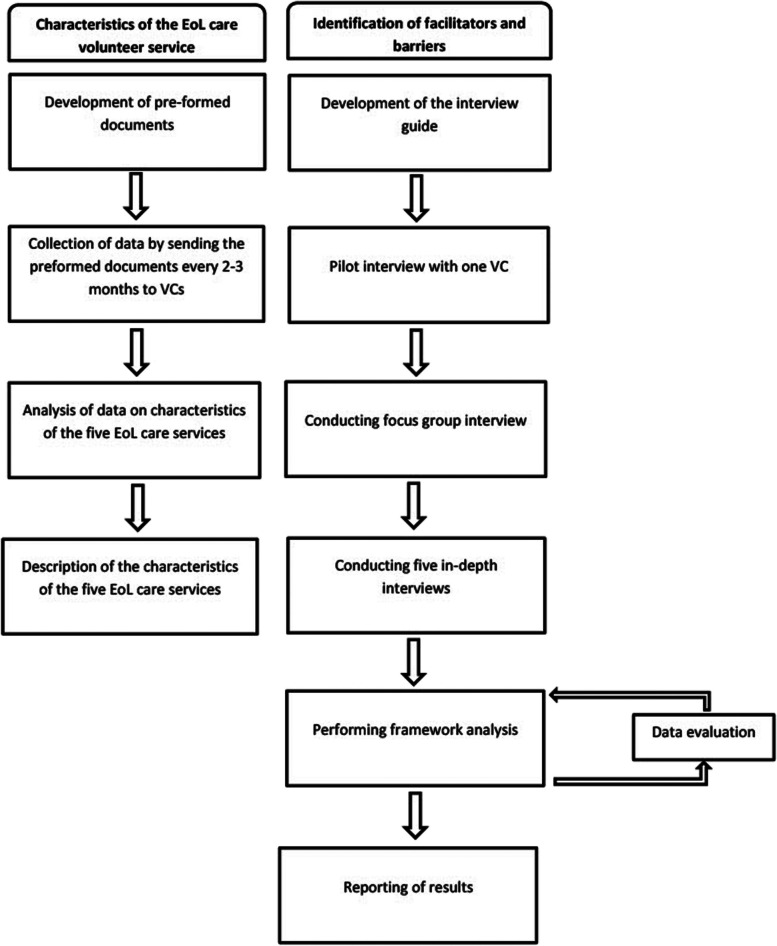
Overview of data collection methods and data analysis

The process of development of the EoL care volunteer services started early 2020. To collect data, the case study methodology according to Yin was applied, using multiple methods and data sources [17]. This case study approach provides the ability to deal with the comparison of different phenomena in complex and context dependent situations [18]. By using multiple data sources, the goal was to increase the validity of the research findings. This method also seemed useful given the international character of the study and therefore to partly compensate for the inability to be on-site. The first type of data collected was descriptive information about the volunteer services, using preformed documents that were shared with the researchers to inform them about the status of the service development in each country at regular intervals. Logbooks were used to report about decisions that were taken regarding the structure of the service. The second type of data collection were the focus group interview [19] and the one-to-one in-depth interviews with VCs [20, 21]. An overview of data collection methods and analysis is provided in Fig. 1.

After careful comparison of implementation theories, the Consolidated Framework for Implementation Research (CFIR) was used as a frame to inspire data collection and analysis [22, 23]. The CFIR identifies factors that influence an intervention’s implementation and includes five major domains, each consisting of a number of constructs: Intervention, Inner setting, Outer setting, Characteristics of individuals, and Implementation process. (Table 3).

**Table 3:** Explanation of the domains of the Consolidated Framework for Implementation Research (CFIR) Model

1. **Intervention:** characteristics of the intervention that is implemented. Constructs within this domain are for example the perceived difficulties of the intervention 2. **Inner setting**: features of the organisation in which the intervention is implemented. Constructs within this domain relate to the implementation climate: the level of priority assigned to the intervention, organisational incentives and the degree to which goals are clearly communicated 3. **Outer setting**: features of the external context or environment, for example relevant external policies and incentives 4. **Characteristics of individuals** involved in the implementation. This domain includes beliefs and knowledge of individuals about the intervention and identification with their organisation and its goals 5. **Implementation process:** strategies used for implementing the intervention, such as planning, executing, reflecting and evaluating	

A semi-structured interview guide was developed using the interview guide tool available on the CFIR website [24]. The CFIR interview guide was reviewed to identify and select questions relevant to the implementation of the volunteer services. A pilot interview was then conducted with one VC using the CFIR interview guide with selected questions. Following this pilot interview, the interview guide was adapted to include self-developed questions to facilitate the interviews. The interview guide is included as a supplementary file.

#### Focus group interview

The face-to-face focus group interview was scheduled in May 2022 as part of a project meeting in a research and education center in Malaga, Spain. One VC from each site and two VCs from the Dutch site participated. At the beginning of the focus group interview, the moderator explained the aim and procedure of the focus group interview and in-depth interviews. She also explained the role of the moderator and the researcher who was present to take field notes during the focus group interview.

During the focus group interview, VCs were asked to list the top three factors that helped or hindered implementation. Each VC then shared their list of factors and the group elaborated on topics that were deemed important. Discussion was facilitated by asking questions from the CFIR interview guide and whether other VCs had similar or different experiences. At the end of the focus group, all VCs were asked to fill in a paper sheet that included a radar chart covering the five domains of the CFIR extended with an additional domain regarding the COVID-19 pandemic. The meaning of the domains and related constructs as included in the interview guide was explained. The VCs were asked to rate to what extent each domain of the CFIR influenced the implementation. The VCs could choose a score between -5 and 5 to rate each domain on the radar chart. A positive score (1 to 5) indicated a positive influence of a specific domain on the implementation of the EoL care volunteer service, while a negative score (-1 to -5) indicated a negative influence of a specific domain on the implementation. For example, a score of 5 indicated an extremely facilitating influence of that domain on the implementation process. The VCs from the Dutch site completed one radar chart together. The duration of the interview was 1 h. The interview was audio recorded and transcribed verbatim.

Both the moderator (AG, PhD) and the researcher (BY, MSc) who took field notes were female. AG is a professor of care ethics by occupation and BY a PhD candidate. Both researchers are by education trained to perform qualitative research.

#### In-depth interviews

To gain deeper insight into the facilitators and barriers for the implementation processes, one semi-structured in-depth interview per site was conducted with either one (the Netherlands, Spain, Slovenia, United Kingdom) or two (Norway) VCs. The in-depth interviews took place two to four months after the focus group interview was conducted. During the in-depth interviews, VCs were asked to give a detailed description of site-specific experiences visualized in the rating of each CFIR domain on the radar chart. Experiences related to their scores on the chart were explored verbally. Questions from the CFIR interview guide were asked for in-depth understanding on how relevant constructs under the domains influenced the implementation. The mean duration of the interviews was 55 min (range: 35—67 min). All in-depth interviews took place via Zoom and were conducted by a female researcher (AG). The interviews were audio recorded and transcribed verbatim.

### Data analysis

Data from the preformed documents and logbooks were analysed within and between sites to get insight into the characteristics of each EoL care volunteer service. Analyses of the focus group and in-depth interview data started by studying the radar charts. Then, the transcripts of the in-depth interviews and the focus group were read to get familiar with the data, focusing on facilitators and barriers for implementation. Data saturation was discussed after the focus group interview and the five in-depth interviews. The domains of the CFIR were used as the theoretically inspired framework to conduct framework analysis [25, 26]. Framework analysis was used because of its structured approach to summarize, compare and contrast data from the different contexts of the sites in relation to the five dimensions of the CFIR model [25].

Then, data of both the focus group interview and in-depth interviews were summarized under each domain for each EoL care volunteer service. Within this framework, facilitators and barriers were specified, and reflection on similarities and differences between sites took place. This resulted in a table per site illustrating the identified facilitators and barriers under each CFIR domain. This table includes facilitators and barriers identified during both the focus group interview and the five in-depth interviews.

Data that were not directly obvious to which domain of the theoretical framework they might belong, were placed in a separate column. After discussion among the authors about whether and which domain of the theoretical framework, if any, would be most appropriate, the data were integrated into the findings as well. This was done by including the data to the table of facilitators and barriers per site.

The first author (BY) performed the framework analysis and the last author (AG) evaluated twice whether the summaries were adequately answering the main research question under each CFIR domain [25]. The last author provided feedback in the analysis document, and the content and focus of the first author’s summaries were discussed in meetings. When AG identified summaries that did not directly answer the research question, such as descriptions of processes and contexts, suggestions were provided to identify and formulate facilitators and barriers as well. In this way, facilitators and barriers were identified together with descriptions of the different contexts and processes in which the facilitators and barriers were experienced by the VCs. This ensured the quality of the summaries during the analysis process. A member check about the written results of the analysis was performed with the VCs for comments or corrections.

## Results

**Fig. 2:**
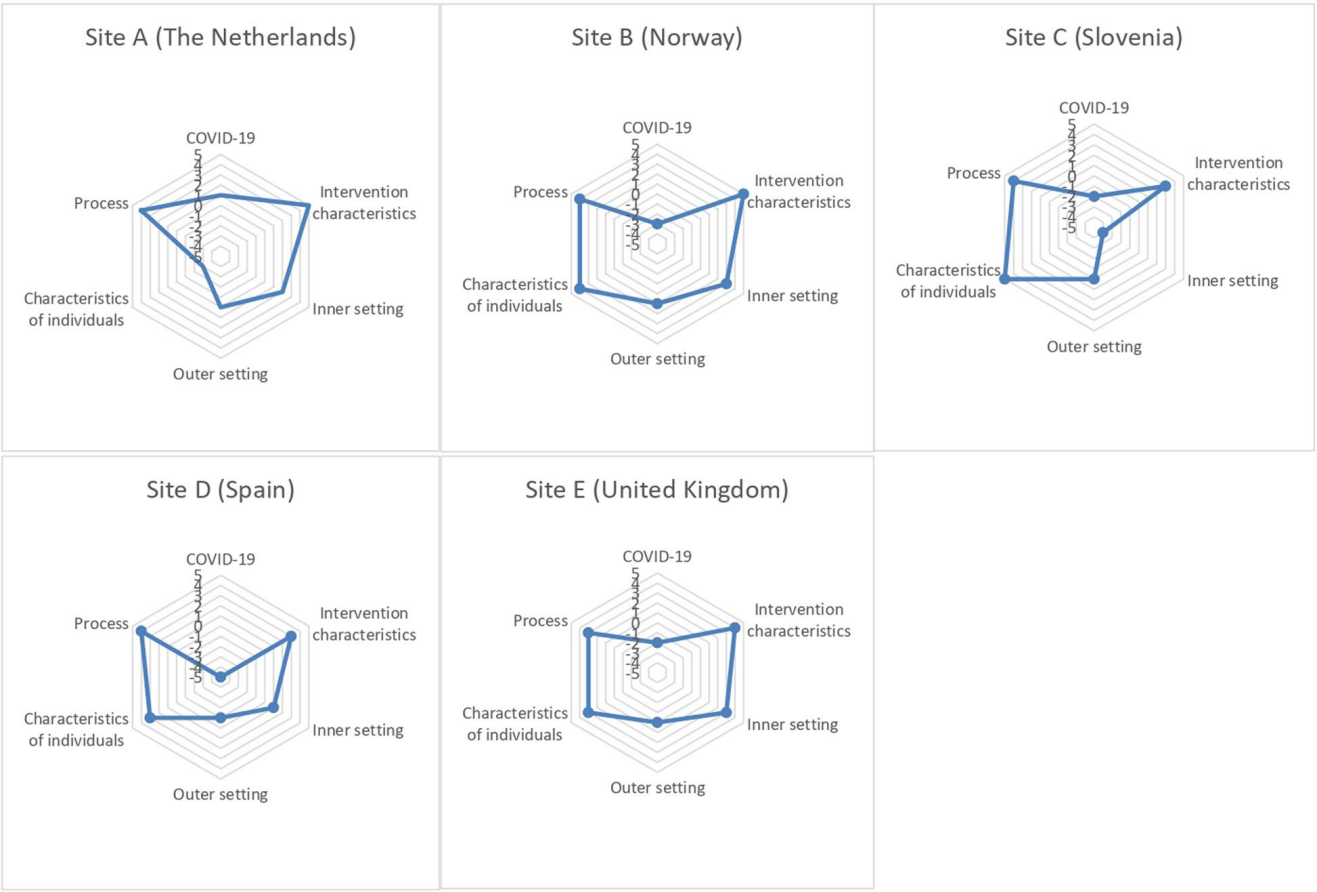
Consolidated Framework for Implementation Research (CFIR) domain scores depicted in radar charts per site

Generic as well as site-specific barriers and facilitators were identified regarding the implementation in the five hospitals. In the following account of the results, pandemic related aspects, which posed a similar significant barrier across all sites, are distinguished from site-specific barriers and facilitators, which provide insight into the different contexts in each site. In addition, an overview of all site-specific facilitators and barriers is provided in the supplementary file. Figure 2 shows the scores for each CFIR domain per site.

### Pandemic related aspects

In all hospitals, the COVID-19 pandemic and associated measures were unpredictable and experienced as an enormous barrier to implementing the EoL care volunteer service (Fig. 1, Supplementary file). One main hindering aspect of the pandemic, across all hospitals, was that volunteers were not allowed access to the hospital for a long period of time as imposed by governmental regulations. It was important to keep the volunteers motivated as the restrictions led to decreased motivation among volunteers who had completed the training and were ready to support patients. Consequently, some volunteers from the site in the UK decided to leave the service. In the site in Spain, possibilities for e-volunteering (e.g. telephone contact) were explored during this period.

Another barrier was that VCs had difficulties reaching healthcare professionals who were under high pressure. As a result, attempts to spread the word about the EoL care volunteer service were delayed or cancelled. Even in between waves of the pandemic, it was hard to promote the service due to the staff feeling exhausted and searching for balance in their departments. Consequently, the services had to be carefully introduced.

*“Also during COVID, healthcare providers from one ward were displaced to another and there were a lot of mixing and stress about this. Then after each wave of COVID back to their original wards, it was stressful for them. I think this was also when they saw it [the volunteer service], they were like “oh ok, one thing more”. Then we slowly started to come one time, then another time. We started to make these promotions with postcards and with meetings. Then we repeated this meeting with managers and repeated this meeting on the ward. So it was a process.” (Coordinator 1, Site C, Slovenia, in-depth interview).*

### Site-specific facilitators and barriers

#### Site A (The Netherlands)

The implementation in the Dutch site was mainly facilitated by positive experiences from the CFIR dimension of process (score 4) and hindered by barriers in the CFIR dimensions of inner setting (score 2) and characteristics of individuals (score -3). The process of implementing was described as a process of learning, reflecting and adapting. The VCs strived to acquire knowledge through their conversations and meetings with others. This facilitated the structure of the service and provided them with new strategies or led to adapting existing strategies. In addition, a facilitator was that healthcare staff, patients and families acknowledged the added value of the service. However, the opinion of some nurses who were skeptical about the service was perceived as a barrier, particularly among nurses with more years of work experience compared to nurses who graduated more recently:

*“The service was easy to tell and then we were waiting, we hear that patients around us are dying and could easily have had volunteers supporting them. Until we learned that we also had to deal with the opinion of the nurse. That was new to us. So if you [the nurses] think: “yes, but it's my patient and I'm here anyway”, they will not call us if they think that way. While they did say what a nice service, how great that this exists, we also immediately resolved by saying that the service is not only for a patient who is alone, but also for a patient with family. So keep it open, just ask us. And if they had an opinion, then I just noticed: “you're not going to call me”.” (Coordinator A1, The Netherlands, in-depth interview)*

Although the existence of a general volunteer service in the hospital was viewed as a facilitator in the CFIR dimension of the inner setting for implementation and recruitment of volunteers, the hierarchical structure within the hospital was experienced as posing a huge barrier. It was a challenge for the VCs to reach healthcare professionals on the wards to disseminate information about the service. Moreover, the working environment in the hospital characterized by shortages of personnel and a large number of flex workers (i.e. nurses) deployed on different wards depending on the needs of the ward, presented an additional barrier. The VCs therefore had to deal with nurses who were not fully engaged with a patient due to their brief presence on each ward.

*“A coordinator cannot get that deep into care. You need an intermediary, in this case, the chaplains. Or a contact person who has been at family meetings, for example. But that [the route] takes a huge amount of time because the route is user-unfriendly: you have to go through a lot of layers, on every part of the day you have to deal with different people, this also differs per ward. Different people all the time, means that you have to explain things all over again. Also, if they have not passed it on to each other properly in the patient file, you get time pressure on the patient. Ideally, you should get rid of some of the layers.” (Coordinator A1, the Netherlands, in-depth interview)*

A barrier regarding characteristics of individuals was to adapt to the diverse opinions held by various stakeholders within the hospital setting. Specifically, the VCs encountered difficulties during conversations about the volunteer service, which tended to move into different directions depending on whether they were held in a group or on a one-to-one basis. Additionally, the VCs had to deal with the views of individuals who joined the project team at a later stage, which impeded the implementation process. With regard to the characteristics of the intervention (score 5), the volunteer training and supervision sessions for volunteers were believed to foster a sense of friendship among the volunteers, which facilitated the continuity of the service.

#### Site B (Norway)

The implementation in the Norwegian site was mainly facilitated by the climate in the inner setting (score 3), positive beliefs about the volunteer service (score 5), and characteristics of individuals (score 4). One main facilitator was the perceived added value of the volunteer service for all stakeholders: the patients, relatives, nurses and physicians, and the hospital management (CFIR domain outer setting, score 2). The VCs considered the volunteer service to be beneficial for all those involved. Although some skeptical nurses worried about volunteers taking away their role, nurses who had established a closer relationship with their patients mostly asked for a volunteer:

*“The third part I think is the nurses and the health care personnel working in the clinic and on the ward. Because it is a relief for them too. And I, what I see in this part is that when they call me to ask if a volunteer can be there, it is usually for a patient that they know very well, they have a relation to the patient and that goes to the feelings of the nurse. He or she they feel that “I know this patient, she is dying; I have a relation with her, I don’t want her to die alone…but I don’t have the time to sit there” and then they call us. I think that is an added value for this.” (Coordinator B1, Norway, in-depth interview)*

The involvement of volunteers during the implementation process was one of the most important facilitators for implementation. The implementation is described as a democratic process in which volunteers are valued for their work and have a say in the decision-making process during the implementation of the service.

*“[…] it is a democratic process, they have been into every decision, and they have discussed every topic around how to fill the role of a volunteer in our hospital so they own the project on the whole. They think about it, they read the information, they discuss how to do this, we change, if they want something changed and it seems like a good thing to do, we change. It is not about anyone’s prestige, it is about doing a good job and they decide how to do a good job in this. (Coordinator B1, Norway, in-depth interview)*

A facilitator related to the nature of the volunteer service was that next to the VCs, healthcare staff also spread the word about the existence of a hospital volunteer service they found valuable.

*“That said, I would say that people who know about this, the department or clinics knowing…they are very positive. Yes, so we have not met anyone saying, “Oh no what’s this, we don’t want this”. They are very positive. People are calling me from unexpected clinics and ask me “Is it true that you have some volunteers who can do this and can do that.” For me that is very positive and kind of self-advertisement. They hear about it from other people.” (Coordinator B2, Norway, in-depth interview)*

#### Site C (Slovenia)

In the Slovenian site, most barriers were identified in the inner setting (score -4). Typical for the Slovenian setting were patients who were not familiar with volunteering. The entire concept of EoL care volunteering had to be introduced in the hospital, requiring considerable effort from the VC and staff. The VC experienced difficulties to spread information to the healthcare professionals. Another barrier was the closure of the palliative care ward due to COVID-19 during implementation. Due to this, the volunteer service had to be adapted in order to be offered at other wards. Nevertheless, regular meetings with different groups of healthcare professionals were identified as a facilitator in this process. In addition, the VC experienced a feeling of trust among healthcare professionals during the hectic periods of the pandemic:

*“And I think during these first years we did a lot of work during the COVID and the healthcare workers got to know us and I think there was an increase in trust also to me and [colleague] and that’s why the volunteer intervention was also more successful because they listened to what we want to say, what we think, what we suggest, and we kind of try new things. It is not easy here to implement something new because patients get many busy schedules. There are many things, which are going on here is a bit hectic, or it is just normal to be hectic with many things happening. “ (Coordinator C1, Slovenia, in-depth interview)*

A facilitator in the outer setting (score 0) was that the volunteer service was considered to be an added value for patients, as the patient population consisted of patients with pulmonary diseases. The VC believed that volunteers could offer support by calming patients but were also aware that patients were vulnerable for infections by volunteers. A facilitator regarding characteristics of the intervention (score 3) was the quality of the training leading to gaining knowledge among the group of volunteers and feelings of enthusiasms. In addition, they felt connected with each other and were motivated, despite the volunteer training sessions taking place online:

“*And I think they have clear motivation why they want to help people at the end of life and this was I don’t know connected together. And they were here, they were available even if they cannot come here or they do not want to come here because of Covid for example, different reasons, but they were mentally here with us. I think this was also important. As a coordinator, it was a good feeling knowing you had someone you could call. They are not just like dropping out but they are here.” (Coordinator C1, Slovenia, in-depth interview )*

With regard to characteristics of individuals (score 5), the VC experienced a lack of support and appreciation towards the volunteer service from the management level. However, the support they received from the head of nurses and physicians turned out be a facilitator. In addition, having a retired nurse among volunteers was a facilitator as she emphasized the importance of such a service to other volunteers and healthcare professionals. In addition, because of her experience with patients with pulmonary problems she could advice on volunteer tasks that could benefit this specific patient population, such as assistance to get fresh air.

#### Site D (Spain)

The implementation of EoL care volunteers in the hospital in Spain was coordinated by two coordinators from the volunteer department of a local hospice. The VCs described the implementation process as a learning process (score 4). Introducing themselves and the volunteer service to the staff in the hospital appeared to be challenging. Different strategies were needed to facilitate communication between volunteers, the volunteer department of the local hospice and the hospital. In addition, barriers in the inner setting (score 1) were the lack of support from the management level of the hospital, and uncertainty about their role in the development and implementation of the service, which delayed the implementation.

*“It was like, you know as [name of hospice organisation] we are a very well-known organisation and it was.. Sometimes it was really difficult to get to a hospital when you are a well-known organisation and.. It was like sometimes the hospital was feeling like we were going to teach them. It was like if we were the best and they were the worst, something strange of the head of the organisation. That part was really difficult.” (Coordinator D1, Spain, in-depth interview)*

A barrier in the outer setting was unfamiliarity with the concept of EoL care volunteering among healthcare staff and patients (score -1). However, the VC undertook activities to engage the wider public and to introduce the volunteer service, for example through the use of social media. This also facilitated recruitment of volunteers.

Although the staff in the hospital had a positive view on EoL care volunteering, a barrier was that staff were not available all the time, and volunteers found it challenging to communicate with them about patients:

*“Sometimes we felt that they [hospital staff] wanted it [the volunteers] not all the time. Only in the time it was useful for them. Let me see if I can explain myself. Our volunteers go there in the afternoon from 5pm to 7pm. So the volunteers started maybe 20 minutes early to get into the hospital [..] But sometimes they felt that was not the best moment for the staff because it was a busy afternoon because they did not have enough time. It was one of the difficult ones because some volunteers were more open or who have more tools they could manage to get more information or let them go and get more information later.” (Coordinator D1, Spain, in-depth interview)*

#### Site E (United Kingdom)

The inner setting (score 3) in the hospital in UK was characterized by a climate in which volunteering is highly valued and welcomed. EoL care volunteers had already been active in the hospital for a longer period. The support from the staff at the palliative care ward towards volunteers turned out to be an important facilitator. Volunteers being valued as part of the teams by the staff was important in the implementation, as they came back again which facilitated the patients to see the value of it. At wards other than the palliative care ward, raising awareness about the service was a challenge as they were not familiar with this type of volunteering. Therefore, it was noted that it took time for healthcare staff to become aware of the volunteer service’s availability to support their patients.

With regard to the characteristics of the service (score 4), there were strict views on the role and boundaries of what a volunteer can or cannot do in the context of end of life. Education and awareness about this among volunteers and staff were identified as facilitators. A good working relationship between the VC, volunteers and staff at the ward was an important facilitator in the CFIR domain of characteristics of individuals:

*“I think something that’s really important that has been positive is the relation between the volunteer service staff and the staff within the palliative care department and the staff in the ward where the volunteers work. Having good communication and good working relationship is really important. And I would say we definitely have that and without that I think that would hinder the implementation and the service as a whole, but we work really well together and can go to each other if we have questions or concerns. […] I think that’s really important, and everyone is aware of what those volunteer role boundaries are and the purpose of the volunteer and what they need support wise in order to succeed in that role.” (Coordinator E1, United Kingdom, in-depth interview)*

## Discussion

This study investigated the facilitators and barriers to the implementation of a novel form of EoL care volunteering: in inpatient hospital settings. Pilots in five hospitals in five European countries were involved. Using the CFIR model, both generic and site-specific barriers and facilitators regarding implementation were identified. Similar influences across sites were the COVID-19 pandemic delaying the implementation process, and the necessity to raise awareness about the new volunteer service due to lack of recognition among hospital staff about the added value of EoL care volunteers. Site-specific facilitators influencing the implementation were the presence of a general volunteer service in the hospital, quality of the volunteer training, and involving volunteers themselves in promoting the service. Education and awareness for healthcare professionals about the role, conceptualization and added value of, and boundaries in interacting with volunteers were also identified as facilitators. Site-specific barriers were struggles with promoting the service in a highly structured setting with many stakeholders, unfamiliarity with the concept of EoL care volunteering in the hospital and negative views among nurses about this source of care in the hospital. Moreover, a lack of support from healthcare professionals and the management, and uncertainty among VCs regarding their role during implementation were also perceived as barriers.

### Complexity of implementing community care

Within the literature, volunteering is considered a unique source of providing community care, in addition to professional and family care at the end of life [6, 9, 27]. However, by incorporating community care (EoL care volunteering) into the highly specialised environment of a hospital, particular challenges and considerations can be expected due to clashes in cultures of care. In all hospitals, implementation of such a service appeared to be a complex and time-consuming process. This was partly caused by the COVID-19 pandemic and the measures restricting access of volunteers to hospitals [28], but mainly also by other barriers related to the inner and outer organisational context, the intervention itself, and characteristics of individuals involved.

In this sense, the findings of the present study fit into the theoretical model of dissemination and implementation of healthcare innovations developed by Greenhalgh and colleagues [29], which served as the foundation for the development of the CFIR model. According to Greenhalgh et al., implementation is viewed as a complex process organised under certain components such as communication and system readiness, while interactions of these components occur within the social, political and organisational context. Using the CFIR model, it was possible to identify why VCs experienced certain barriers and facilitators during the implementation process, such as nurses expressing positivity about the service while being convinced that caring for patients was their own job.

### Inner setting

Although it is challenging to evaluate the impact of socio-cultural site-specific aspects on the implementation of the five services, unfamiliarity with EoL care volunteering in the hospital and negative views among nurses about volunteers in all sites, required serious time and communicative efforts of all VC’s. In site A (the Netherlands), this was even more complicated since the VCs had no direct links to clinical staff and thus encountered challenges to reach staff from various levels at the clinical wards. In addition, due to the working culture among healthcare professionals (i.e. flex workers) in this hospital, the VCs had to deal with nurses who had little information about patients and changing staff, as also demonstrated in another study about experiences of volunteers [12]. In contrast, it was found that nurses in the Norwegian site who had established a good relationship over time with their patients mostly asked for a volunteer. These findings indicate the importance of analysing the interaction of implementation components with social, political and organisational contexts, including working conditions of available staff, for understanding the differences in utilization of the volunteer service in different hospital settings. A clear conceptualization of “being there” may prevent a medical, nursing or task-oriented understanding of the contribution of volunteers to care in hospital contexts [6] and may increase constructive collaboration with professionals.

It should also be noted that a good patient-nurse relationship may introduce a risk for selection bias and unequal access to the volunteer service. For instance, patients who may experience difficulties to establish a relationship with nurses, for example due to language barriers, may be seen as less likely to be supported by a volunteer than those who have no or less difficulty establishing relationships. Themed sessions about equity or unconscious bias for volunteer coordinators and hospital staff is recommended to ensure equal access to volunteer support. In addition, recruiting a diverse group of volunteers may help minimize the risk of selection bias and unequal access [30].

One finding was that among all sites, only the Dutch VCs experienced challenges during conversations with individuals who had negative opinions about the volunteer service. This may be due to the prevailing idea in the Netherlands that death should not take place in the hospital, but at home or in community places such as a hospice. This meant that patients had a short length of stay in the hospital and therefore there was a narrow window of time to offer the volunteer service. However, it has been suggested that bringing community care to the hospital not only helps to fill the social gaps when a patient lacks visiting family, but might also lead to better transfers of patients back to a hospice or to their home [31]. In addition, even for short periods of admission to the hospital, volunteers in healthcare may have the capacity to improve patients’ experiences of care [32]. This may be even more important in the light of a growing population with palliative care needs [33].

### Involvement of important individuals

The findings of this study indicate that implementation of EoL care volunteering in the hospital setting requires involvement of stakeholders in a way that enables medical and EoL care volunteering to co-exist. On the one hand, it is important to address the views of nurses about the role and boundaries of volunteers while emphasizing the knowledge that volunteers do not replace the role of paid staff [11]. On the other hand, volunteers should not only be informed, guided and enabled to perform their role [34], but also involved in decision-making during implementation. In our study, an organisational facilitator was that volunteers in site B (Norway) were from the beginning involved in decision-making about approaches and how to work the service. This approach may imply that EoL care volunteers may be viewed as equal members of the healthcare team [11]. A previous study has suggested that despite volunteers being regularly informed on how patient care was organised, they still had no decision-making power and were not regularly invited to contribute to how patient care was organised [35]. It is recommended to further explore how trust and equal partnerships between volunteers and paid staff can be built and sustained [1]. These factors are modifiable and should therefore be considered in order to improve EoL care volunteering in hospital settings [36].

### Strengths and limitations

This study has several strengths. To our knowledge, this is one of the first studies to present findings on implementation aspects of EoL care volunteering in the hospital setting. Previous studies have mainly focused on experiences of volunteers, providing insight on the training needs of volunteers, and the difficulties and benefits of fulfilling the role of a volunteer in the hospital setting [12, 13, 37]. Another strength of our study is that the study group was able to collect qualitative data in an international context. In addition, collection of data was done by combining different methods such as a focus group, in-depth one-to-one interviews and data from preformed documents and logbooks.

A limitation of this study is that due to the international nature of the project, it was not feasible to conduct ethnographic research in the sites. Observation of (non)verbal interactions may provide more in-depth knowledge about the implementation process. However, the CFIR is based on relevant implementation theories in a variety of disciplines [22] and offered a clear structure for data collection and analysis. Further research is needed to investigate how innovations involving EoL volunteering should be adapted to the context of the hospital, especially in light of the trend that many people die in hospitals in middle- and high- income countries [38]. Another limitation is that the interviews were conducted with VCs who were in varying stages of implementation due to the impact of the pandemic. Consequently, the complete range of experiences for those who had started the implementation of the volunteer service shortly before the interviews took place not have been fully captured. Therefore, there may be additional facilitators and barriers that were not presented in the findings. Nevertheless, this study provides insights into the factors that contribute or hinder implementation of EoL care volunteer services in hospitals, highlighting areas for further investigation.

One limitation of the study may be related to the potential bias resulting from the VCs being part of an international project. On the one hand, it is plausible that the VCs may have been particularly motivated, leading to positive attributions of feelings and experiences regarding the process. On the other hand, they may have felt pressure to aim for a successful implementation of the service, potentially leading to negative attributions of meaning to the implementation process. It is possible that VCs have overlooked certain barriers or facilitators. However, it is important to note that a substantial number of both facilitators and barriers across all dimensions of the CFIR framework was identified. Therefore, it is not expected that this has affected the findings.

## Recommendations 

Based on the findings of this study, it is recommended to increase awareness and provide education among healthcare professionals regarding the role and benefits of EoL care volunteers in the hospital setting. This can be achieved through training programs addressing the conceptual core of EoL care volunteering organised by collaboration of VCs and healthcare professionals in hospitals. Regular communication and research about the value and cost-effectiveness of EoL care volunteering in the hospital respectively is also needed [11]. In addition, further research should explore effective strategies for promoting EoL care volunteering in hospitals and understanding the cultural and contextual factors that influence the implementation of such services. Such research could involve a multi-stakeholder approach to gain insights from healthcare professionals from the management level to frontline staff, volunteers, patients, and their families.

### Supplementary Information

**Supplementary Material 1. **

## Data Availability

Possibilities for sharing data can be discussed upon request, by contacting the corresponding author (BY).

## Abbreviations

CFIR: The Consolidated Framework for Implementation Research
VC: Volunteer coordinator
EoL: End of life
NL: The Netherlands
NO: Norway
SI: Slovenia
ES: Spain
UK: United Kingdom
