# Supplementary material for: Facilitators and barriers of implementing end-of-life care volunteering in a hospital in five European countries: the iLIVE study
Source: BMC Palliat Care. 2024 Apr 2;23:88. doi: 10.1186/s12904-024-01423-5 (PMC10985898; doi:10.1186/s12904-024-01423-5)
Supplement: Supplementary file 1 — Supplementary Material 1. [file 12904_2024_1423_MOESM1_ESM.docx]

**Supplementary file: Facilitators and barriers of implementing End-of-Life care volunteering in a hospital in five European countries: The iLIVE Study.**

***Facilitators and barriers per site according to the domains of the Consolidated Framework for Implementation Research (CFIR).***

| **SITE A - THE NETHERLANDS** | |  |
| --- | --- | --- |
| **Domain** | **Main findings** | |
| Intervention Characteristics | ***Facilitators:***   - It was easy to explain the content of the service, positive reactions when presenting the service. - Use of social media to promote the volunteer service.   ***Barriers:***   - Use of the service was challenging: it was not clear to staff at the eight participating wards how to ask for a volunteer. - The opinion of some nurses who were skeptical about the service. Nurses who think it is their own job to care and be there for the patients themselves, will not call for a volunteer. - Persons in the project team who were involved in the volunteer training did not enroll patients who could be offered a volunteer, although they met many of these patients and their families. | |
| Inner setting | ***Facilitators:***   - Having a general volunteer service in the hospital, meaning that there was a coordinator, general hospital training for volunteers, a routine in terms of administration and basic reimbursement for participating volunteers. - An intermediary (such as a chaplain) who can facilitate connection with clinical staff and recruit patients can be helpful.   ***Barriers:***   - Hierarchy in the hospital and the challenge to reach all the organisational layers for the VC who was not involved in clinical care at the wards. An intermediary such as a chaplain was needed. - When a patient wanted a volunteer, it had to be organized quickly because they were not staying long in the hospital (often a day or two). - Flex workers who worked all over the hospital and did not know the patients very well. | |
| Outer setting | ***Facilitators:***   - Presenting the volunteer service to other hospitals generated interest and motivated the VC. - Involving and asking volunteers how they were thinking about the service. - Conducting a small questionnaire at the clinical wards, to make a profile per ward about their needs and expectations regarding the volunteer service in order to inform volunteers what they can or cannot do.   ***Barriers:***  None | |
| Characteristics of individuals | ***Facilitators:***  None  ***Barriers:***   - The VC’s had to take into account how to communicate depending on talking individually with someone or in a group. - A member of the project team who had close cooperation with clinical care teams, but had an introverted character which did not help to spread the word about the service to other wards and hierarchical levels in the organization. - Colleagues who joined the team later and had a very strong opinion about the volunteer service, delaying the process; whereas the VC expected and preferred them to be part of the process of building on what had been achieved. | |
| Process | ***Facilitators:***   - The “Train the Trainer” training in Liverpool meeting was very useful and gave guidelines to the VC. - The training of volunteers within the Dutch hospital led to a feeling of being a family among the group of volunteers.   ***Barriers:***   - Not having a clear division of tasks within the project team and lack of accountability of project members who were involved in daily patient care and support. - Implementing the service required a lot of learning, changing, adapting and reflecting about decisions and ideas on how to organize the volunteer service and promote it. | |

| **SITE B - NORWAY** |  |
| --- | --- |
| **Domain** | **Main findings** |
| Intervention Characteristics | ***Facilitators:***   - The service was added on to an already existing general volunteer service. - Healthcare staff who found the service valuable spread the word about the volunteer service. - Working together with the communication department facilitated sharing and communicating online about the volunteer service.   ***Barriers:***   - It was difficult for the VC to offer the volunteer service properly; she would like to offer it as long as the patient needed it, which was difficult to organize sometimes. - The opinion of nurses who were skeptical about the service. |
| Inner setting | ***Facilitators:***   - The management had a very positive view on the volunteer service.   ***Barriers:***   - It was difficult to get some promotion starting up. |
| Outer setting | ***Facilitators:***   - The project team members found the volunteer service to be of added value for all stakeholders (patients, relatives, nurses, physicians, management). They believed it will meet their needs. - Nurses who had a good relationship with patients mostly asked for a volunteer. - Volunteering in hospitals is not a topic anywhere in the country except for Oslo and Bergen. The VC’s thought they can be an example for other hospitals.   ***Barriers:***   - Some staff in the hospital were skeptical about the volunteer service. |
| Characteristics of individuals | ***Facilitators:***   - Good communication between the two VC’s. They were able to see things from both sides due to their study background and work experience (communication background vs. palliative care background) and put them together.   ***Barriers:***  None |
| Process | ***Facilitators:***   - Involving volunteers during the implementation process with decision-making. Volunteers owned the service, in a democratic way. - It was believed that a meeting with the ward leaders at the hospital where the VCs can talk for 5 minutes about the service would be good for implementation. - “Train-the-trainer” training, meeting in Liverpool (iLIVE research project) was good to start with and gave motivation. - Volunteers have been informed very well about the project.   ***Barriers:***   - COVID-19 restrictions. |

| **SITE C - SLOVENIA** |  |
| --- | --- |
| **Domain** | **Main findings** |
| Intervention characteristics | ***Facilitators:***   - The European training for volunteers and educational tools were helpful for the VC in order to have a good background of knowledge about end of life volunteering.   ***Barriers:***   - Having no palliative care ward anymore due to COVID-19, which led to adapting the volunteer service to be offered at other wards, and searching for patients in their last phase of life. |
| Inner setting | ***Facilitators:***   - Increasing trust between the VC and healthcare professional (HCP) during intensive collaboration during COVID-19.   ***Barriers:***   - Patients were not familiar with volunteering, therefore many strategies were needed to promote the service. - There was no private hospital rooms for patients to have intimate conversations with volunteers. - Hectic care setting made it hard to figure out how to implement the service. |
| Outer setting | ***Facilitators:***   - The patient population which often had problems with breathing, therefore it was good that volunteer could calm patients.   ***Barriers:***   - The hospital management and healthcare providers perceived the volunteers as a barrier because having contact with a volunteer could mean that patients with pulmonary diseases were extra vulnerable to be infected by having contact with volunteers. - Due to lack of appreciation from the management, there was no comparing with other institutions. |
| Characteristics of individuals | ***Facilitators:***   - Having a retired nurse in the group of volunteers. She brought enthusiasm and explained the importance of the volunteer service to other volunteers. - Receiving support from the management of head nurse, also from the director of physicians. - Connecting with social worker who helped with development of the (research) intervention group. - Using clear and direct communication to the volunteers, but also supportive when the training was finished. - Good collaboration with colleague to coordinate the service. - Volunteers’ recognition of the importance to be involved in the project. - Being a part of something bigger, i.e. the European research project, helped implementation by motivating volunteers.   ***Barriers:***   - A lack of support from the management. There was not a feeling that the management appreciated the service. |
| Implementation process | ***Facilitators:***   - Meeting with different groups of healthcare professionals regularly. - The content of the volunteer training led to volunteers having a clear motivation to support patients at the end of life, and feeling connected. - The ability to have close contact with a group of experts due to the international project. - Meetings with other international volunteers increased motivation of the volunteers. - Using promotional material, writing an article for a newspaper about the volunteer service. - VCs were able to be creative in how to lead the implementation because there were open ends next to the common grounds of the research project.   ***Barriers:***   - The VC’s struggled to spread information to healthcare professionals. Many strategies were needed. - The human factor that it needed time that the new role of someone coming to the hospital is accepted and validated. - Volunteers were often stopped by healthcare staff asking what they are doing, because healthcare staff often did not recognize volunteers or were not aware of the volunteer service. |

| **SITE D - SPAIN** |  |
| --- | --- |
| **Domain** | **Main findings** |
| Intervention Characteristics | ***Facilitators:***   - Training “Train the Trainer” (iLIVE research project) was very good and useful.   ***Barriers:***   - The VC’s and volunteers were not part of the hospital, but linked to a local hospice. Different strategies were needed to introduce themselves and the service, and for the communication. |
| Inner setting | ***Facilitators:***  None  ***Barriers:***   - There was a lack of support from the management of the hospital, which also appeared to feel some level of competition with the VC’s’ organisation. - It was not clear what the role of the management was in the development and implementation of the service. |
| Outer setting | ***Facilitators:***   - Healthcare staff were open to volunteers.   ***Barriers:***   - There was unfamiliarity with the concept of end-of-life volunteering among patients and general public. - One other organization felt competitive. But did not experience pressure from that. |
| Characteristics of individuals | ***Facilitators:***   - Healthcare staff had positive views on volunteering. - The VC’s and volunteer had a good relationship. - Volunteers were prepared for (the challenges of) working with staff in the hospital environment.   ***Barriers:***   - Staff were not available all the time. Volunteers found it challenging to communicate with them. - It was complicated for the VC’s when volunteers talked about their challenges in the hospital. The VC’s were aware that they cannot always change the situation, but this was tiring. |
| Process | ***Facilitators:***   - Taking note of everything, viewing the process as a learning process as VC’s. Evaluating at the same time. - Having a positive attitude during the process, also when challenges arise. - Engagement of the wider general public in talking about and introducing the service. Development of campaigns can help. - Informing the staff about the role of volunteers. - Having enough time to prepare the project. - Use of the 10-steps model for implementation (European Core Curriculum) made the coordinators think about things they had not thought about before.   ***Barriers:***   - Lack of communication with the other VC’s (iLIVE research project). |

| **SITE E - UNITED KINGDOM** | |  |
| --- | --- | --- |
| **Domain** | **Main findings*** | |
| Intervention Characteristics | ***Facilitators:***   - Making sure that everyone is aware and educated about boundaries of the volunteer role. - Having strict boundaries about what a volunteer can and cannot do.   ***Barriers:***  None | |
| Inner setting | ***Facilitators:***   - Volunteering is welcomed in the hospital and high on the agenda. - An atmosphere in which the work of volunteers is valued and they are seen as part of the team.   ***Barriers:***   - Healthcare staff not being familiar with this type of volunteering on wards other than the palliative care ward. Raising the profile and awareness about the service was therefore challenging. | |
| Outer setting | ***Facilitators:***  None  ***Barriers:***  None | |
| Characteristics of individuals | ***Facilitators:***   - Good working relationship between volunteers and staff at the departments. - Good communication about the volunteer service.   ***Barriers:***  None | |
| Process | ***Facilitators:***   - Training of volunteers was very much appreciated by volunteers; it led to a feeling of group cohesion.   ***Barriers:***  None | |

* Due to more years of experience in implementing an EoL care volunteer service in this site, and a recent organizational change, fewer facilitators and barriers were identified compared to other sites.

**Interview guide**

**iLIVE Volunteer study (WP3) – Facilitators & Barriers implementation**

***Background***

An objective of the Volunteer Study (iLIVE-VS) is to assess the implementation of the iLIVE-VS within participating hospitals in order to generate knowledge about enabling and hindering factors. Other hospitals that want to implement an End of Life Volunteer service will benefit from this information.

The questions asked during the focus group interview and individual interviews were formulated based on the domains of the CFIR described on [Updated CFIR Constructs – The Consolidated Framework for Implementation Research (cfirguide.org)](https://cfirguide.org/constructs/).

Several relevant constructs and questions (to the implementation of the volunteer service) were selected in the CFIR interview guide, available on <https://cfirguide.org/evaluation-design/qualitative-data/>, to include in the current interview guide.

***Introduction***

In this focus group interview/in-depth interview, we would like to talk about the development of the volunteer service you have been coordinating so far. We are interested in the facilitating factors and barriers that influenced the implementation of the volunteer service in the hospital. We would like to ask questions about your experiences with implementing the service so far. Please feel free to tell your story with your accents.

**Focus group interview**

**1. Question:** When considering the implementation process of bringing in the volunteer service in your hospital, which three influences were significant according to your experiences? These three influencing factors can be positive or negative, i.e. hindering or facilitating the implementation process. Please take some time to think about it and write it down if necessary. *[To facilitate discussion, it is encouraged to ask questions from the CFIR interview guide below for in-depth understanding per domain]*

*[After explanation of the 5 dimensions of CFIR with selected constructs (find below), and instructions]:*

**Radar chart**

**2. Question:** To what extent did the domains influence the implementation of the volunteer service in a positive or negative way?

- Please indicate a score between – 5 (strong negative influence) and + 5 (strong positive influence) for each domain.

**In-depth interview**

During the focus group interview you have rated the influence of each CFIR domain on the implementation of the volunteer service in your hospital on a radar chart. In this interview, we would like to gain more in-depth insight how the domains and related constructs (find below) have influenced the implementation of the volunteer service.

**Question:** Can you please clarify why you rated the domains with that specific score?

*[It is encouraged to ask questions from the CFIR interview guide below for in-depth understanding per domain]*

**Consolidated Framework for Implementation Research (CFIR)**

*The following questions under the selected constructs (in bold) can be used to ask specific questions interviews related to the domains, during the focus group interview and in-depth. Also use as prompt questions is encouraged.*

Intervention Characteristics

**Adaptability**

1. What kinds of changes or alterations do you think you will need to make to the volunteer service so it will work effectively in your setting?
   - - With respect to the original plan
     - With respect to the COVID-19 pandemic and restricting measures
2. Are there components that should not be altered?
   - Which ones should not be altered?

**Evidence Strength & Quality**

1. *In a healthcare setting, influential stakeholders may include influential and well-respected clinicians.*

What do influential stakeholders think of the volunteer service?

**Complexity**

1. Do you think that the volunteer service is a complicated intervention?

- If yes, how complicated is the volunteer service?
- Why? What makes it complicated or not?

**Design Quality & Packaging**

1. What supports, such as online resources, marketing materials, or a toolkit, are available to help you implement and use the volunteer service?
   - Have you used:
     - Learning Lab
     - 10 steps model
     - European Core Curriculum (ECC)
     - Questionnaire on motivation volunteers
     - Staff survey to identify expectations of staff from the volunteer service
2. How have these materials influenced (successful) implementation in your setting?

Outer Setting

**Patient Needs & Resources**

1. To what extent were the needs and preferences of the individuals served by the volunteer service considered when deciding to implement the volunteer service?
   - Can you describe specific examples?
2. How well do you think the volunteer service will meet the needs of the individuals served by your organization?
   - In what ways will the volunteer service meet their needs?
3. How do you think the individuals served by the volunteer service will respond to the volunteer service?
4. What barriers will the individuals served by the volunteer service face to participating in the volunteer service?
5. Have you gained information from participants regarding their experiences with the volunteer service?
   - What are their perceptions of the volunteer service?

**Peer Pressure**

1. Can you tell me what you know about any other hospitals in your country that have implemented the volunteer service or other similar programs?
   - How has this information influenced the decision to implement the volunteer service?
2. To what extent are other wards within your hospital implementing the volunteer service?
   - How does that affect support for implementing the volunteer service in your own setting?
3. To what extent would implementing the volunteer service provide an advantage for your hospital compared to other organizations/hospitals in your area?

Inner Setting

**Structural Characteristics**

1. How did the infrastructure of your hospital (social architecture, age, maturity, size, or physical layout) affect the implementation of the volunteer service?
   - How will the infrastructure facilitate/hinder implementation of the volunteer service?
   - How will you work around structural challenges?
2. What kinds of infrastructure changes will be needed to accommodate the volunteer service?
   - For example regarding: changes in scope of practice? Changes in formal policies? Changes in information systems or electronic records systems? Other?

**Networks & Communications**

1. Can you describe your working relationships with your colleagues?
   - With colleagues (in your ward)?
   - With colleagues in other wards?
2. Are meetings, such as staff meetings, held regularly?
   - Do you typically attend?
   - Who typically attends?
   - How often are the meetings held?
   - How helpful are these meetings?

**Culture**

1. How do you think your organization's culture (general beliefs, values, assumptions that people embrace) will affect the implementation of the volunteer service?

**Implementation Climate**

1. What is the general level of receptivity in your organization to implementing the volunteer service?
   - Why?

**Tension for Change**

1. Is there a strong need for this volunteer service?
   - Why or why not?

**Compatibility**

1. How well does the volunteer service fit with your values and norms and the values and norms within the organization?

**Relative Priority**

1. How important do you think it is to implement the volunteer service compared to the other priorities?

**Leadership Engagement**

1. What kind of support or actions can you expect from leaders in your hospital to help make implementation successful?
   - Who are these leaders? How do attitudes of different leaders vary?
   - What types of barriers might they create?

Characteristics of Individuals

**Knowledge & Beliefs about the Intervention**

1. How do you feel about the volunteer service being used in your setting?
   - Do you have any feelings of anticipation? Stress? Enthusiasm? Why?

**Self-efficacy**

1. How confident are you that you will be able to successfully implement the volunteer service?
   - What gives you that level of confidence (or lack of confidence)?
2. How confident do you think your colleagues feel about implementing the volunteer service?
   - What gives them that level of confidence (or lack of confidence)?

Process

**Planning**

1. What have you done (or what do you plan to do) to get a plan in place to implement the volunteer service?
2. What role has your plan for implementation played during implementation?
   - Was it used to guide implementation of the volunteer service?
   - Was the plan shared/reviewed with other stakeholders? How regularly?

**Formally Appointed Internal Implementation Leaders**

1. Who did lead implementation of the volunteer service?
   - What attributes or qualities does this person have that makes them an effective leader of this implementation?

**Key Stakeholders**

1. What is your communication or education strategy for getting the word out about the volunteer service?

- What materials/modes/venues do you plan to use?

COVID-19

1. How did the Covid-19 pandemic influence the implementation process?

**Supplemental figure 1.** Timetable of the project, including timeslots of data collection and analysis.
